# Supplementary material for: Level of Physical Activity in Pregnant Populations from Different Geographic Regions: A Systematic Review
Source: J Clin Med. 2022 Aug 8;11(15):4638. doi: 10.3390/jcm11154638 (PMC9369818; doi:10.3390/jcm11154638)
Supplement: Supplementary file 1 [file jcm-11-04638-s001.zip › jcm-1784687-supplementary.pdf]

Supplementary

Methods

Search strategies

Table S1. PICOS framework.

PICOS	DEFINITIONS
**Population**	(“pregnancy” OR “maternal” OR “antenatal” OR “pregnant”)<br>(“health” OR “wellbeing”)
**Interventions**	(“physical activity” OR “exercise” OR “training”)<br>(“barriers” OR “enablers” OR “access”)
**Comparison**	(“physical activity” OR “exercise” OR “training”)<br>(“barriers” OR “enablers” OR “access”)
**Outcome**	(“physical activity” OR “exercise” OR “training”)<br>(“maternal outcome” OR “pregnancy outcome”)
**Study design**	(“observational” OR “cohort” OR “qualitative”)<br>NOT<br>(“randomized clinical trial” OR “RCT”)

PubMed:

Article publication period: (2006-2021)

1. (“physical activity” OR “exercise” OR “training”)
2. (“barriers” OR “enablers” OR “access”)
3. (“pregnancy” OR “maternal” OR “antenatal” OR “pregnant”)
4. (“health” OR “wellbeing”)
5. (“maternal outcome” OR “pregnancy outcome”)
6. (“observational” OR “cohort” OR “qualitative”)
7. (“randomized clinical trial” OR “RCT”)
8. 1 and 2
9. 1 and 2 and 3
10. 1 and 3 and 4
11. 1 and 2 and 5
12. 1 and 3 and 6
13. 1 and 3 and 6 not 7
14. 1 and 2 and 5 and 6
15. 1 and 2 and 5 and 6 not 7

16. Limit 15 to full text articles
17. Limit 16 to English, Spanish and Portuguese

Web of Science:

Article publication period: (2006-2021)

1. (“physical activity” OR “exercise” OR “training”)
2. (“barriers” OR “enablers” OR “access”)
3. (“pregnancy” OR “maternal” OR “antenatal” OR “pregnant”)
4. (“health” OR “wellbeing”)
5. (“maternal outcome” OR “pregnancy outcome”)
6. (“observational” OR “cohort” OR “qualitative”)
7. (“randomized clinical trial” OR “RCT”)
8. 1 and 2
9. 1 and 2 and 3
10. 1 and 3 and 4
11. 1 and 2 and 5
12. 1 and 3 and 6
13. 1 and 3 and 6 not 7
14. 1 and 2 and 5 and 6
15. 1 and 2 and 5 and 6 not 7
16. Limit 15 to Highly Cited Papers
17. Limit 16 to English, Spanish and Portuguese
18. Limit 15 to Search by Countries/Regions

SportDiscus with full text:

Article publication period: (2006-2021)

1. (“physical activity” OR “exercise” OR “training”)
2. (“barriers” OR “enablers” OR “access”)
3. (“pregnancy” OR “maternal” OR “antenatal” OR “pregnant”)
4. (“health” OR “wellbeing”)
5. (“maternal outcome” OR “pregnancy outcome”)
6. (“observational” OR “cohort” OR “qualitative”)
7. (“randomized clinical trial” OR “RCT”)
8. 1 and 2
9. 1 and 2 and 3
10. 1 and 3 and 4
11. 1 and 2 and 5
12. 1 and 3 and 6
13. 1 and 3 and 6 not 7
14. 1 and 2 and 5 and 6
15. 1 and 2 and 5 and 6 not 7
16. Limit 15 to English and Spanish

17. Limit 15 to type of the document “Article”
18. Limit 15 to Search by Countries/Regions

<u>Scopus:</u>

Article publication period: (2006-2021)

1. (“physical activity” OR “exercise” OR “training”)
2. (“barriers” OR “enablers” OR “access”)
3. (“pregnancy” OR “maternal” OR “antenatal” OR “pregnant”)
4. (“health” OR “wellbeing”)
5. (“maternal outcome” OR “pregnancy outcome”)
6. (“observational” OR “cohort” OR “qualitative”)
7. (“randomized clinical trial” OR “RCT”)
8. 1 and 2
9. 1 and 2 and 3
10. 1 and 3 and 4
11. 1 and 2 and 5
12. 1 and 3 and 6
13. 1 and 3 and 6 not 7
14. 1 and 2 and 5 and 6
15. 1 and 2 and 5 and 6 not 7
16. Limit 15 to English and Spanish and Portuguese
17. Limit 16 to type of the document “Article”
18. Limit 17 to Source Type “Journal”
19. Limit 18 to Publication stage “Final”
20. Limit 16 to Search by Countries/Regions
